# *IGFBP3* Methylation Is a Novel Diagnostic and Predictive Biomarker in Colorectal Cancer

**DOI:** 10.1371/journal.pone.0104285

**Published:** 2014-08-15

**Authors:** Lucia Perez-Carbonell, Francesc Balaguer, Yuji Toiyama, Cecilia Egoavil, Estefania Rojas, Carla Guarinos, Montserrat Andreu, Xavier Llor, Antoni Castells, Rodrigo Jover, C. Richard Boland, Ajay Goel

**Affiliations:** 1 Division of Gastroenterology, Department of Internal Medicine, Charles Sammons Cancer Center and Baylor Research Institute, Baylor University Medical Center, Dallas, Texas, United States of America; 2 Gastroenterology Department, Hospital Clinic, CIBERehd, IDIBAPS, University of Barcelona, Barcelona, Spain; 3 Department of Pathology, Hospital General Universitario de Alicante, Alicante, Spain; 4 Research Unit, Hospital General Universitario de Alicante, Alicante, Spain; 5 Gastroenterology Department, Hospital del Mar, Barcelona, Spain; 6 Department of Medicine and Cancer Center, University of Illinois at Chicago, Chicago, Illinois, United States of America; 7 Unidad de Gastroenterología, Hospital General Universitario de Alicante, Alicante, Spain; Howard University, United States of America

## Abstract

**Background and Aim:**

Aberrant hypermethylation of cancer-related genes has emerged as a promising strategy for the development of diagnostic, prognostic and predictive biomarkers in human cancer, including colorectal cancer (CRC). The aim of this study was to perform a systematic and comprehensive analysis of a panel of CRC-specific genes as potential diagnostic, prognostic and predictive biomarkers in a large, population-based CRC cohort.

**Patients and Methods:**

Methylation status of the *SEPT9, TWIST1, IGFBP3, GAS7, ALX4 and miR137* genes was studied by quantitative bisulfite pyrosequencing in a population-based cohort of 425 CRC patients.

**Results:**

Methylation levels of all genes analyzed were significantly higher in tumor tissues compared to normal mucosa (p<0.0001); however, cancer-associated hypermethylation was most frequently observed for *miR137* (86.7%) and *IGFBP3* (83%) in CRC patients. Methylation analysis using the combination of these two genes demonstrated greatest accuracy for the identification of colonic tumors (sensitivity 95.5%; specificity 90.5%). Low levels of *IGFBP3* promoter methylation emerged as an independent risk factor for predicting poor disease free survival in stage II and III CRC patients (HR = 0.49, 95% CI: 0.28–0.85, p = 0.01). Our results also suggest that stage II & III CRC patients with high levels of *IGFBP3* methylation do not benefit from adjuvant 5FU-based chemotherapy.

**Conclusion:**

By analyzing a large, population-based CRC cohort, we demonstrate the potential clinical significance of *miR137* and *IGFBP3* hypermethylation as promising diagnostic biomarkers in CRC. Our data also revealed that *IGFBP3 hyper*methylation may serve as an independent prognostic and predictive biomarker in stage II and III CRC patients.

## Introduction

Colorectal cancer (CRC) is one of the most common cancers in Western countries, and is the second leading cause of cancer-related deaths in adults [1]. Accumulating evidence indicates that epigenetic changes play an essential role in the pathogenesis of CRC. Aberrant DNA methylation represents one of the most studied epigenetic alteration, and it is well understood that tumor suppressor genes are frequently silenced by methylation of CpG islands which commonly reside in the 5′ regions of approximately half of all human genes [2], [3], [4]. Transcriptional silencing of tumor suppressor genes has emerged as an important step in the step-wise process of colorectal carcinogenesis. Epigenetic alterations, particularly DNA hypermethylation, can be used for the early detection of premalignant lesions, including adenomatous polyps in the colon, and is present in the non-neoplastic tissues adjacent to adenomatous polyps and cancers in the colon [5], [6]. In addition to their use as diagnostic biomarkers, epigenetic changes show promise as determinants of cancer prognosis, as well as biomarkers for response to specific chemotherapies [7], [8].

In CRC, systematic genome-wide approaches have identified several genes that show tumor-specific promoter hypermethylation that can potentially be developed into clinically relevant diagnostic, prognostic and predictive biomarkers. In the context of CRC, several such biomarkers have recently been described as potentially promising diagnostic biomarkers, including: *SEPT9* (Septin 9), a member of the septin family involved in cytokinesis and cell cycle control [9], [10], [11]; *ALX4* (Homeobox protein aristaless-like 4), a transcription factor involved in skull and limb development [12], [13]; *TWIST1* (Twist homolog 1), an antiapoptotic and pro-metastatic transcription factor [14], [15]; *IGFBP3* (Insulin-like grown factor binding protein 3), a member of the insulin-like growth factor binding protein family [16]; *GAS7* (growth arrest-specific 7), which plays a putative role in neuronal development [17], [18]; and *miR137*, a non-coding microRNA which is embedded in a CpG island [17], [19], [20], [21]. However, to date, none of these methylation markers have undergone a systematic validation in a large cohort of CRC patients to fully ascertain their potential as clinically useful diagnostic, prognostic or predictive biomarkers. For this reason, we aimed at exploring the diagnostic, prognostic and predictive value of *SEPT9, TWIST1, ALX4, IGFBP3, GAS7*, and *miR137* promoter hypermethylation in a large, well-characterized, population-based CRC cohort. Furthermore, we examined associations between the methylation status of individual markers and their combination with the clinicopathological features in these primary CRCs, and for the first time report that *IGFBP3* hypermethylation is a promising diagnostic and predictive biomarker in CRC patients.

## Material and Methods

### Patients

This study included 425 CRC patients that were enrolled as part of the Epicolon-I project, which is a population-based trial of CRC as described previously [22], [23], [24]. Since this study aimed to determine the prognostic and predictive potential of methylation biomarkers, the patient specimens included in this study were randomly selected from a previously described cohort of patients for whom follow-up data were available [7], [25], [26]. Demographic, clinical, and tumor-related characteristics of probands, as well as a detailed family history, were obtained using a previously established questionnaire [22]. Clinicopathological and molecular features of patients included in those studies are described in **Table S1.** The study was approved by the ethics committee of all participating hospitals in the EPICOLON cohort (Hospital 12 de Octubre, Madrid; Hospital Clinic, Barcelona; Hospital Clínico Universitario, Zaragoza; Hospital Cristal-Pinor, Complexo Hospitalario de Ourense; Parc de Salut Mar, Barcelona; Hospital Donostia, CIBERehd, University of Country Basque, San Sebastian; Hospital General Universitario de Alicante; Hospital General de Granollers; Hospital General de Vic; Hospital General Universitario de Guadalajara and Fundación para la Formación e Investigación Sanitarias Murcia; Hospital General Universitario de Valencia), and written informed consent was obtained from each patient. The promoter methylation status of six genes (*SEPT9, TWIST1, IGFBP3, GAS7, ALX4* and *miR-137*) was analyzed in all 425 CRC patients, as well as in normal colonic mucosa from 21 healthy individuals with a normal colonoscopy.

### DNA extraction and bisulfite modification

Genomic DNA was extracted from paraffin-embedded tumor tissue (FFPE) specimens in the Epicolon-I study. Under supervision of the study pathologist, tissue sections were carefully examined, tumor-enriched regions identified and carefully dissected for DNA extraction. Following paraffin removal by xylene, DNA was isolated using QiaAmp DNA Mini kits, as per the manufacturer's instructions (Qiagen, Valencia, CA). The resultant genomic DNA was modified with sodium-bisulfite using the EZ Methylation Gold Kit (Zymo Research, Orange, CA).

### DNA Methylation analysis

Bisulfite pyrosequencing was performed for quantitative methylation analysis as previously described (**Figure S1**) [7], [25], [26]. Primers used were designed using the PyroMark 1.0 design software, and pyrosequencing assays were run on the bisulfite-modified DNA (**Table S2 and Information S1**). Mean percentage methylation for all the CpG sites in each assay was calculated for each gene/marker. Methylation cut-off values were determined for each marker based on the average methylation levels observed in normal colonic mucosa from healthy individuals +2 standard deviations. Additional confirmatory methylation analysis for *IGFBP3* was also undertaken by a quantitative MSP (qMSP) assay for the promoter/exon1 CpG island using the primers and PCR conditions as previously described [27].

### CpG Island Methylator Phenotype (CIMP) and Microsatellite Instability (MSI) status

The CIMP status of the CRC samples from the Epicolon-I cohort was previously determined using bisulfite pyrosequencing of the CIMP markers *CACNAG1*, *SOCS1*, *RUNX3*, *NEUROG1*, and *MLH1*, as reported previously [7]. A CRC was considered CIMP-positive if at least 3/5 promoters were methylated [7]. Microsatellite instability (MSI) testing was performed using BAT26 and NR24 quasimonomorphic markers as previously described [28]. Tumors were classified as MSI-positive when either marker was mutated, and were deemed microsatellite stable (MSS) when neither marker demonstrated any evidence for genetic instability.

### *BRAF* mutation

Presence of the *V600E BRAF* mutation in CRC samples was detected using TaqMan probes and an ABI Prism 7500 sequence detection system (Applied Biosystems, Foster City, CA), with allelic discrimination, as previously described [29].

### Statistical Analysis

Continuous variables are reported as mean + standard deviation (SD), while categorical variables are cited as frequency or percentages. Statistical differences of baseline characteristics between groups were analyzed using the χ^2^ test for categorical data, followed by application of Yates' correction and the Mann-Whitney U test for quantitative data analysis. The primary outcomes of this study were overall survival (OS) and disease-free survival (DFS). Analyses of both outcomes were performed for the entire CRC cohort, and separately in the subset of stage II and III CRC patients in order to specifically evaluate the effect of methylation status on the prognosis and response to adjuvant chemotherapy. OS was defined as the time from enrollment to death, and DFS was defined as the time from enrollment to death from any cause or the first relapse. Data on OS and DFS were censored at 1100 days from the date of cancer diagnosis. CRC tumors were categorized into high and low methylation status groups using Receiver Operating Characteristics curve (ROC) analysis. Kaplan-Meier analysis was performed to estimate the distributions of DFS and OS in stage II and III patients. Survival distributions were compared using the log rank test. A multivariate analysis for determination of hazard ratios for death or tumor recurrence was performed using several variables, including, lymph node metastasis, TNM stage, MMR status, tumor differentiation grade, age and adjuvant chemotherapy. Further analyses were performed using Cox's proportional hazards regression in a stepwise manner to test the effect of methylation status of a given gene/marker with DFS and its interaction with chemotherapy. Hazard ratios and 95% confidence interval (95% CI) for death were computed using Cox survival modeling. All reported p values are two sided, and p values <0.05 were considered significant. All calculations were performed using SPSS 10.0 or GraphPad Prism 4.0 statistical software.

## Results

In our series of 425 CRC cases, the mean ± SD age of the patients was 70.4 +−11 years, and there were more male patients than females (252/425; 60.3%). Of the 425 tumors, 117 (27.9%) were located in the proximal colon, 151 (36.2%) were in the distal colon, and 150 (35.9%) were located in the rectum. Approximately 12.8% of the cases were stage I, 33.7% stage II, 39.7% stage III, and 13.7% stage IV tumors. We found that 35/425 (8.2%) CRCs were MSI, 90/303 (29.7%) were CIMP-positive, and 21/191 (10.9%) harbored a somatic V600E mutation in the *BRAF* gene. As expected, the CIMP-positive phenotype was more frequently associated with MSI and the presence of *BRAF* V600E mutation (22.3% vs. 2.3%, p<0.0001 for the MSI phenotype, and 31% vs. 1.5%, p<0.0001 for the *BRAF* V600E mutation).

### Diagnostic significance of methylation markers in CRC

**Figure 1:**
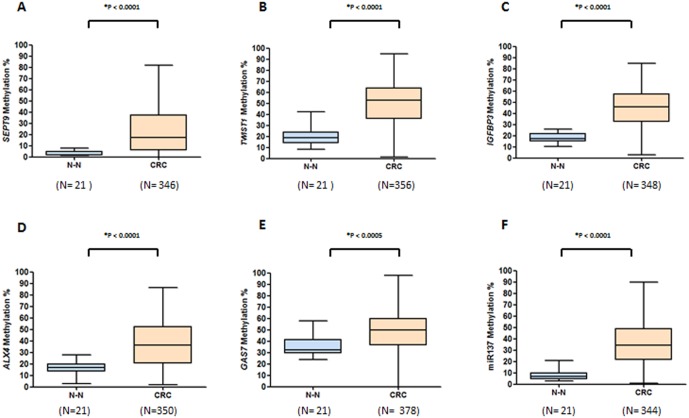
Comparison between levels of methylation in primary tumors vs. normal colonic mucosa. Bisulfite pyrosequencing results for methylation of *SEPT9* (A), *TWIST1* (B), *IGFBP3* (C), *ALX4* (D), *GAS7* (E) and *miR137* (F) genes comparing normal mucosa from healthy controls (N–N) and primary tumors from CRC patients.

**Figure 2:**
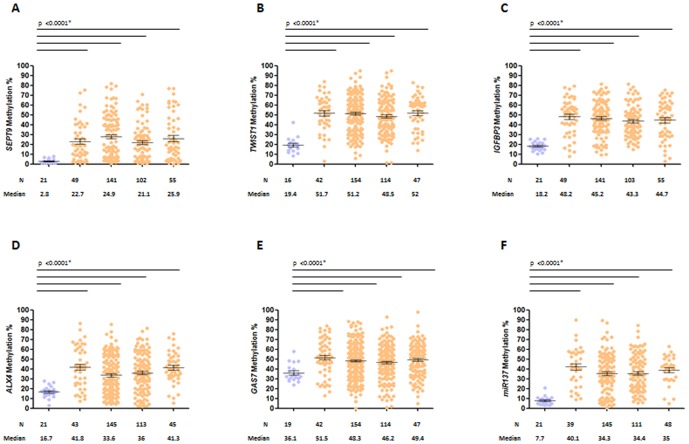
Levels of methylation in normal colonic mucosa and crc tumors from all tnm stage patients. Bisulfite pyrosequencing results of methylation of *sept9* (a), *twist1* (b), *igfbp3* (c), *alx4* (d), *gas7* (e) and *mir137* (f) comparing normal mucosa from healthy patients (n-n) and primary tumors from crc patients of all tnm stages i–iv. n (number of subjects); median of methylation (%).

The mean methylation levels for each gene in primary CRC tissues were: 23.8% for *SEPT9*, 50.5% for *TWIST1*, 44.9% for *IGFBP3*, 50.2% for *GAS7*, 36.5% for *ALX4*, and 35.3% for *miR137*. As shown in **Figure 1**, methylation levels of each gene were significantly higher in CRC versus normal mucosa from healthy subjects (*p*<0.0001 to *p*<0.0005 for all comparisons). These results demonstrate the cancer-specificity for hypermethylation of these genes and establish a rationale for their exploitation as potential diagnostic biomarkers for CRC, considering that all 6 markers demonstrated hypermethylation in all stages of CRCs (**Figure 2**).

In order to assess the performance of each methylation marker individually for CRC diagnosis, methylation results were analyzed as a categorical variable. Accordingly, each gene was classified as methylated when mean methylation levels were higher than 7.1% for *SEPT9*, 35.7% for *TWIST*, 27.5% for *IGFBP3*, 53.5% for *GAS7*, 28.5% for *ALX4*, and 11.9% for *miR137*. Based upon these analyses, the most frequently methylated markers in CRC tissues were the *miR-137* gene (302/344, 87.7%), followed by *IGFBP3* (289/348, 83%), *TWIST1* (269/356, 75.6%), *SEPT9* (244/346, 70.5%), *ALX4* (214/350, 61.1%), and *GAS7* (164/378, 43.3%). To further assess whether a combination of markers would further enhance the diagnostic accuracy of the assay, we analyzed combination marker panels and found that the *miR137*+*IGFBP3* combination yielded an diagnostic accuracy of 86%, followed by *TWIST1*+*IGFBP3* (82.7%) and *TWIST1*+*miR137* (78.5%). Furthermore, we found that the combination of *miR137*+*IGFBP3*+TWIST1 methylation had the highest diagnostic accuracy, 92%, as shown in **Table 1** **.**

**Table 1:** Performance Characteristics Of Methylation Markers For The Identification Of Colorectal Cancer.

Gene(s)	Sensitivity (%)	Specificity (%)	PPV (%)	NPV (%)	Accuracy (%)
***SEPT9***	70.5	95.2	99.6	16.4	65.8
***TWIST1***	75.6	93.8	99.6	14.7	69.3
***IGFBP3***	83.0	100.0	100.0	26.3	83.0
***GAS7***	42.9	94.4	99.4	7.3	37.3
***ALX4***	61.1	100.0	100.0	13.4	61.6
**miR137**	87.8	90.5	99.3	31.1	78.3
**miR137+** ***IGFBP3***	95.5	90.5	99.3	59.4	86.0
***TWIST1+IGFBP3***	92.2	90.5	99.3	45.2	82.7
***TWIST1*** **+miR137**	92.8	85.7	98.9	45.0	78.5
***miR137+IGFBP3+TWIST1***	98.7	93.3	99.3	87.5	92.0

PPV, Positive Predictive Value; NPV, Negative Predictive Value.

### Clinicopathological features associated with methylation biomarkers

**Figure 3:**
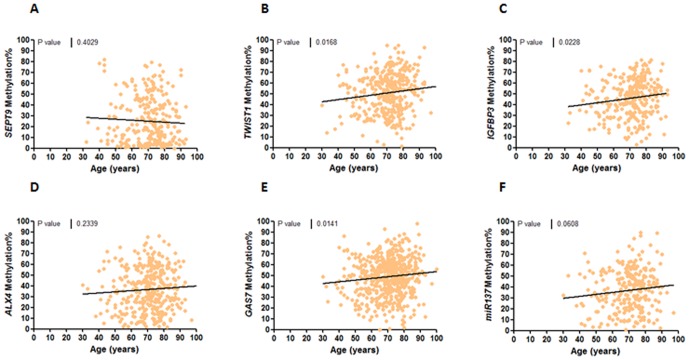
Correlation between primary tumor methylation and age of the crc patients. linear regression analysis comparing tumor methylation of *sept9* (a), *twist1* (b), *igfbp3* (c), *alx4* (d), *gas7* (e) and *mir137* (f) vs age of crc patients (years).

We next investigated the relationship between various clinicopathological and molecular features and their association with aberrant methylation of all six genes analyzed in this study. The clinicopathological variables included age, gender, TNM staging and tumor location, and the molecular factors included the MMR status, CIMP phenotype and the *BRAF* mutational status. Tumor location and TNM stages did not associate with the methylation status of any of the gene markers. Curiously, *SEPT9* methylation was significantly associated with male gender (p<0.05; **Table S3**). When methylation was analyzed as a continuous variable, we observed a trend for higher methylation of *TWIST1*, *IGFBP3, ALX4*, and *GAS7* (p<0.05) and older patients with CRC; however, we did not find a positive correlation between normal colonic mucosa methylation in these genes and age in healthy individuals (**Figures 3** **&** **4**).

**Figure 4:**
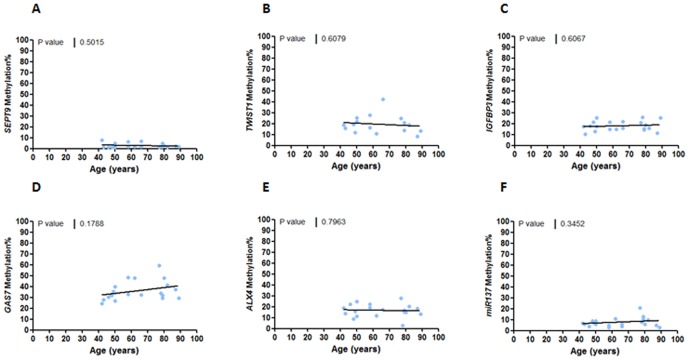
Correlation between colon mucosa methylation and age of healthy controls. linear regression analysis comparing tumor methylation of *sept9* (a), *twist1* (b), *igfbp3* (c), *alx4* (d), *gas7* (e) and *mir137* (f) vs age of the healthy control patients (years).

With regards to molecular associations, *TWIST1* methylation levels were higher in MSS compared to MSI CRCs (51% vs 40.3%; p<0.05) (**Table 2**). Methylation of four genes, *ALX4, IGFBP3, GAS7*, and *miR137*, was significantly correlated with CIMP-positive tumors (p<0.001). Furthermore, as shown in **Table 2**, CRCs with *ALX4* and *IGFBP3* methylation were more frequently associated with a somatic *BRAF* V600E mutation (13.5% vs. 1.8%, p<0.05 for *ALX4*, and 12.4% vs. 0%, p<0.05 for *IGBFP3*).

**Table 2:** Molecular features associated with gene methylation in crc patients.

Genes	MSI Status (% methylation)			CIMP Status (% methylation)			*BRAF Mutation (% methylation)*		
	Mean + SD			Mean + SD			Mean + SD		
	MSI	MSS	P-value*	Positive	Negative	P-value*	Mutated	WT	P-value*
***SEPT9***	20.9 + 18.9	24.5 ± 20.6	NS	24.5 + 20.5	25.3 + 21.4	NS	17.1 + 14.3	23.4 + 20.8	NS
***ALX4***	34.6 + 20.2	36.6 + 19.5	NS	44.5 ± 17.6	35.8 + 18.3	<0.0001	51.8 + 6.2	35.8 + 17.7	<0.001
***TWIST1***	40.3 + 51.1	51.0 + 18.5	< 0.05	53.4 ± 17.2	50.2 + 17.2	NS	52.3 + 12.6	51.6 + 17.4	NS
***IGFBP3***	47.5 + 18.7	44.8 + 17.3	NS	51.4 ± 17.2	43.5 + 17.2	<0.001	54.5 + 13.1	43.7 + 17.7	< 0.05
***GAS7***	49.9 + 17.9	48.3 + 17.0	NS	56.0 + 10.1	48.6 + 15.5	<0.0001	54.1 + 13.7	49.1 + 15.9	NS
***miR137***	38.4 + 21.6	35.1 + 17.9	NS	43.1 ± 15.4	34.4 + 17.7	<0.001	42.2 + 14.4	35.4 + 17.7	NS

*Evaluated with student's -t test

SD: Standard deviation; l; mmr: mismatch repair; msi: microsatellite instability; mss: microsatellite stable. Cimp: cpg island methylator phenotype; wt: wild type; ns: no significant.

### Aberrant hypermethylation of genes and its influence on CRC patient prognosis

The median follow-up for the series of 425 patients included in this study was 1268 days (3.4 years; range 0–2204 days). At the end of the follow-up period, 169 patients had died (39.7%), and the median follow-up for this group was 671±513 days (1.8±1.4 years). Information regarding the cause of death was available in 99 patients; 67.7% (67/99) died due to complications of tumor progression, 18.2% (18/99) died due to complications of chemotherapy, and 14.1% (14/99) succumbed to death from other causes, including post-operative complications. Tumor recurrence was observed in 116/370 patients (31.3%), with a median time duration of 644±448 days (1.7±1.2 years) post-surgery.

**Figure 5:**
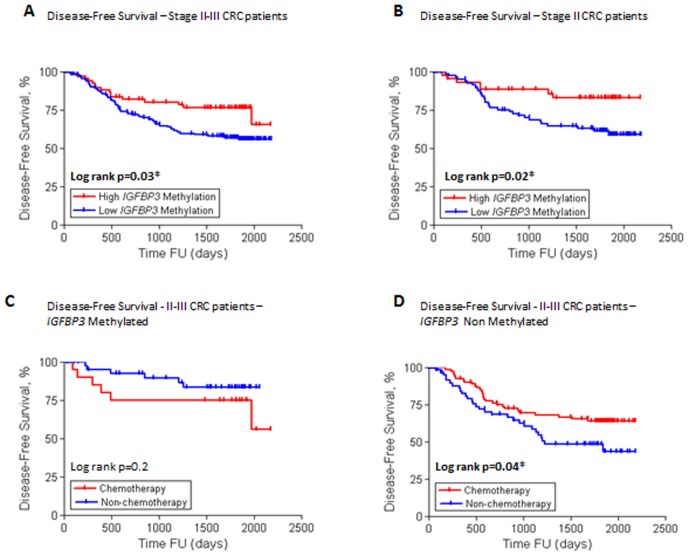
Relationship between igfbp3 methylation and prognostic and predictive response in stage ii and iii crc patients. (a) Disease-free survival of patients with stage ii and iii crc, according to *igfbp3* methylation status (high methylation, n = 73, (30.3%) low methylation, n = 168, (69.7%). (b) Disease-free survival of patients with stage ii disease, according to *igfbp3* methylation (high methylation, n = 45, (32.4%); low methylation, n = 94, (67.6%). (c) Disease-free survival of patients with stage ii and iii disease, in high *igfbp3* methylation tumors based on adjuvant chemotherapy (chemotherapy, n = 20, (22.5%); no chemotherapy, n = 69, (77.5%). (d) Disease-free survival of patients with stage ii and iii disease, in low *igfbp3* methylation tumors according to adjuvant chemotherapy (Chemotherapyc n = 83, (52.7%); NO Chemotherapy, n = 74, (47.2%).

To determine the prognostic significance of the methylation status for each gene, all CRCs were categorized into two groups based on their methylation levels (high versus low), and the dose–response relationship between the degree of methylation and event-free survival was examined by ROC curve analysis. Overall, high levels of methylation of the *GAS7* (prognosis cut-off value = 52.62%; high-methylation: 43.3%, low-methylation: 56.7%; χ^2^ p = 0.03) and *ALX4* genes (prognosis cut-off value = 25.8%; high-methylation: 67.1%, low-methylation: 32.9%; χ^2^ p = 0.005) associated with poor DFS in CRC patients (data not shown). In contrast, low levels of *IGFBP3* methylation demonstrated an association with significantly worse DFS according to unadjusted analysis (prognosis cut-off value = 53.1%; high-methylation: 30.9%, low-methylation: 69.1%; χ^2^ p = 0.01; **Figures 5A and B**). Following multivariate Cox regression analysis where multiple variables were analyzed, only low levels of *IGFBP3* methylation emerged as an independent risk factor for poor DFS in stage II and III CRC patients (HR = 0.49, 95% CI: 0.28–0.85, p<0.01). Furthermore, the methylation status of the *IGFBP3* gene was the only statistically significant risk factor for stage II CRC, after adjustment for other prognostic factors such as age, adjuvant 5-FU (5-Fluorouracil) chemotherapy or MSI status (HR = 0.28, 95% CI: 0.12–0.70, p = 0.006; **Table 3**).

**Table 3:** Multivariate analysis of disease-free survival based on *IGFBP3* promoter methylation in stage II and III CRC patients.

Stage II and III CRC Patients	Hazard Ratios (HR)	95% CI	P-value
**Tumor size (T3/4 vs. T1/2)**	1.479	0.46–4.69	0.5
**Lymph Node metastasis (Positive vs. Negative)**	1.605	1.01–2.54	**0.04**
**MMR Status (MSI vs. MSS)**	0.739	0.26–2.06	0.5
**Differentiation Grade (Poor vs. Well/Moderate)**	0.86	0.34–2.16	0.7
***IGFBP3*** ** Methylation (High vs. Low)**	0.479	0.26–0.87	**0.01**
**Stage II CRC Patients**	**Hazard Ratios (HR)**	**95% CI**	**P-value**
**Age (> Median vs. < Median)**	0.774	0.36–1.70	0.5
**Serosal invasion (Yes vs. No)**	1.485	0.62–3.51	0.3
**Chemotherapy (Yes vs. No)**	0.61	0.27–1.36	0.2
**MMR Status (MSI vs. MSS)**	0.612	0.13–2.73	0.5
**Differentiation Grade (Poor vs. Well/Moderate)**	0.548	0.20–1.47	0.2
***IGFBP3*** ** Methylation (High vs. Low)**	0.28	0.12–0.70	**0.006**

CI: Confidence interval; mmr: mismatch repair; msi: microsatellite instability; mss: microsatellite stable.

### Aberrant gene hypermethylation and its influence on patient survival following chemotherapy treatment

Chemotherapeutic treatment was given to 257/425 patients (60.5%), mainly using 5FU+leucovorin (236; 91.8% of patients). Among the entire cohort, 315 patients (74.1%) had either a stage II or III disease, and 155 (49.2%) of these received 5FU-based adjuvant chemotherapy. Only 9 stage II and III patients received other chemotherapeutic treatments, and were excluded from further analysis. After a median follow-up of 1221 days (3.3 years), 92 (31.2%) patients with stage II and III showed tumor recurrence, and by the end of the follow-up period, 101 (34.2%) patients from these groups had died.

Adjuvant chemotherapy provided a significant improvement in OS in stage II and III CRC patients compared to patients who did not receive chemotherapy (chemotherapy: 49.1%, no-chemotherapy: 50.9%; χ^2^ p = 0.0001). These differences in OS were also present in multivariate analysis which controlled for age, gender, lymph node metastasis, MMR, and CIMP phenotype status (HR = 0.55, 95% CI: 0.36–0.85, p = 0.007).

Interestingly, among all 6 methylation markers investigated, only *IGFBP3* methylation levels were predictive of the response to 5FU-based chemotherapy. According to our results, patients with low *IGFBP3* methylation levels had longer OS (p = 0.0007) and DFS (p = 0.05) when they received chemotherapy. Conversely, in patients with high *IGFBP3* methylation, adjuvant chemotherapy did not improve OS (p = 0.4) or DFS (p = 0.3; **Figure 5C and D**). Using multivariate Cox regression analysis (**Table 4**), in patients with low-*IGFBP3* methylation, adjuvant chemotherapy independently predicted better OS (HR = 0.49, 95%CI: 0.29–0.80, p = 0.004); however, it did not significantly influence DFS (p = 0.08). On the other hand, MMR status was the only variable that independently predicted OS (p = 0.05) and DFS (p = 0.04) in patients with high *IGFBP3* methylation (**Table 4**). In the patient cohort treated with 5-FU adjuvant chemotherapy, *IGFBP3* methylation status did not independently predict OS and DFS. In contrast, DFS was positively affected by *IGFBP3* methylation status in patients who did not receive adjuvant chemotherapy (HR = 0.41, 95%CI: 0.18–0.91, p = 0.02).

**Table 4:** Multivariate analysis of disease-free survival and overall survival in stage ii and iii crc patients and *igfbp3* promoter methylation status.

	DFS			OS		
	HR	95%CI	P-value	HR	95%CI	P-value
**High-** ***IGFBP3*** ** Methylation Group**						
**Age (>65, vs. <65)**	1.697	0.66–4.35	0.3	1.218	0.49–3.02	0.7
**Gender (Male vs. Female)**	1.802	0.91–3.55	0.08	1.457	0.77–2.74	0.2
**MMR Status (MSI vs MSS)**	0.371	0.14–0.98	**0.04**	0.391	0.14–1.02	**0.05**
**Lymph Node Metastasis (positive vs. negative)**	0.666	0.31–1.41	0.3	0.98	0.59–1.66	0.9
**CIMP (positive vs. negative)**	1.141	0.57–2.26	0.7	1.443	0.74–2.82	0.3
**Adjuvant Chemotherapy (yes vs. no)**	0.721	0.35–1.47	0.4	0.52	0.25–1.05	0.07
**Low-** ***IGFBP3*** ** Methylation Group**						
**Age (>65 vs. <65)**	0.995	0.45–2.16	0.9	1.135	0.66–1.92	0.6
**Gender (Male vs. Female)**	0.897	0.6–1.34	0.6	0.901	0.6–1.34	0.6
**MMR Status (MSI vs. MSS)**	0.65	0.29–1.33	0.2	0.593	0.27–1.26	0.2
**Lymph Node Metastasis (positive vs. negative)**	1.372	0.91–2.05	0.1	1.429	1.05–1.93	0.02
**CIMP (positive vs. negative)**	0.828	0.48–1.41	0.5	0.844	0.49–1.43	0.5
**Adjuvant Chemotherapy (yes vs. no)**	0.655	0.41–1.05	0.08	0.489	0.29–0.80	**0.004**

DFS: Disease-free survival; os: overall survival; hr: hazard ratio; ci : confidence interval; mmr: mismatch repair; msi: microsatellite instability; mss: microsatellite stable; cimp: cpg island methylator phenotype.

## Discussion

In the recent years, a number of promising gene methylation-based diagnostic biomarkers including *SEPT9, TWIST 1, IGFBP3, GAS7, ALX4*, and *miR137* have been proposed for the early detection of colorectal neoplasia. However, the majority of these markers have been confined to initial small-scale discovery studies, and have not been developed into clinical practice as they have not been systematically validated in large, independent cohorts of CRC patients. In view of this gap in knowledge, we designed this study to confirm the diagnostic potential of these biomarkers, and to ascertain their prognostic and predictive values in CRC. In addition, we were interested in examining the associations between aberrant hypermethylation of these genes and clinicopathological features by analyzing a large, well-characterized, population-based cohort of CRC patients.

Based on our quantitative methylation analysis, all six genes interrogated fulfilled the criteria for potential clinical use as diagnostic biomarkers of CRC. These criteria included: a) the methylation of each gene occurred in a tumor-specific manner; b) the methylation levels of each gene in neoplastic tissues were significantly higher than in normal colonic mucosa; and c) none of the genes showed an age-dependent increase in DNA methylation in normal colonic mucosa [30], [31]. Methylation levels of each gene allowed us to classify colorectal tissues as normal or neoplastic. Among all markers investigated, *IGFBP3* and *miR137* methylation levels showed the highest diagnostic accuracy as individual gene markers for the identification of primary CRCs (83% and 78.3%, respectively). This diagnostic accuracy was further improved by combining the methylation status of two gene markers; in particular, the methylation status of *IGFBP3+TWIST1* or *IGFBP3*+*miR137* had the highest accuracy values of 86% and 82.7% respectively, with sensitivity and specificity values > 80%.

We investigated the molecular features associated with tumor methylation levels of these genes. This was of particular interest as molecular classification based on MSI and CIMP status has become increasingly important [32] for its prognostic [33], [34], [35] and predictive role in CRC patients [7], [34], [36]. In this study, four of the six genes were positively associated with the CIMP phenotype (*ALX4, IGFBP3, GAS7*, and *miR137*). Furthermore, a positive correlation between *BRAF* V600E mutation and methylation of the *ALX4* and *IGFBP3* genes was associated with CIMP-high in CRC [37], [38]. As expected, methylation of the *ALX4* and *IGFBP3* genes was associated with the presence of the *BRAF* gene mutation. *TWIST1* methylation levels were higher in patients with MMR-proficient tumors compared to MSI tumors; however, in our cohort, only a small proportion of CRCs were MMR-deficient (5%), and the correlations between MMR status and methylation markers may have been lost when analyzing the group as a whole.

In stage II and III CRC patients who have undergone potentially curative resection, prognosis depends on disease recurrence, which is primarily associated with distant metastasis. In addition, the benefit from adjuvant chemotherapy, especially for stage II CRC patients has remained an area of controversy and chemotherapeutic resistance remains a major problem in these patients. Therefore, the discovery of novel prognostic and predictive biomarkers that can help identify stage II and III patients that are at high risk of recurrence and metastasis, may improve the current strategy for CRC patient stratification and therapy management. To the best of our knowledge, no previous study has examined the relationship between methylation of the *SEPT9, IGFBP3, TWIST1, GAS7, ALX4*, and *miR137* genes with prognosis in patients with CRC, as well as the ability of these biomarkers to predict response to 5-FU-based adjuvant chemotherapy. Based upon our results in this large, well-characterized population-based cohort, stage II and III CRC patients with low levels of *IGFBP3* promoter methylation had poorer OS and DFS compared to those with high levels of DNA hypermethylation. With regards to response to chemotherapy, when 5-FU based adjuvant chemotherapy was administered to stage II and III CRC patients, only those patients with low levels of *IGFBP3*-methylated tumors were positively affected, resulting in longer DFS and OS times, indicating that the benefit of chemotherapy was limited to this group of CRC patients. Curiously, this effect of *IGFBP3* methylation was independent of CIMP status. Nevertheless it is important to point out that although *IGFBP3* methylation status emerged as the only independent factor that predicted poor DFS probability among stage II CRC patients, additional independent clinical studies are required to prove that *IGFBP3* methylation levels in CRC could represent a potential routine test for prognostication of CRC patients, and in improving the management of patients with localized disease in combination with current clinicopathological and molecular tools.

**Figure 6:**
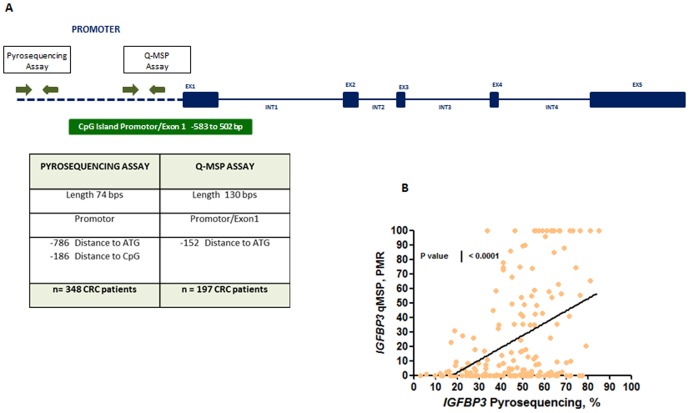
Comparison between methylation levels within igfbp3 promoter in crc tissues. (a) Schematic overview of the insulin-like grown factor binding protein 3 (igfbp3) gene. exons are represented by blue boxes, introns with blue lines, and the promoter region by blue dashed lines. the *igfbp3* gene has one cytosine-phosphate-guanine (cpg) island at in the promoter region and exon1 underscoring the potential of modulating gene expression by means of cpg hypermethylation, and this is represented by a green box. we performed two different assays covering both cpgs islands (indicated by green arrows) using two quantitative methods: pyrosequencing and qmsp in order to correlate methylation levels of these two locations. (b) Linear regression analysis comparing *igfbp3* methylation levels by pyrosequencing (% methylation) in 348 patient samples and qmsp (pmr) assays in crc tissues (n = 197).

Other studies have observed a relationship between high levels of *IGFBP3* methylation and poor clinical outcome in lung and ovarian cancers [39], [40], and more recently in CRC patients [27]. Discrepant reports using methylation markers may be due to the use of different methodologies and the analysis of different sequence locations within CpG Islands. In this study, we used bisulfite pyrosequencing for methylation analysis, which is a reliable quantitative approach for robust DNA methylation analysis compared to the conventional Methylation Specific PCR (MSP), which lacks the quantification provided by bisulfate pyrosequencing [27], [39], [40]. In addition, to further ensure the accuracy of our methylation results, we also conducted a quantitative-MSP (qMSP) analysis within the CpG island region previously studied in CRC [41]. As shown in **Figure 6**, we confirmed that our bisulfite pyrosequencing methylation results were positively correlated to the results obtained within the CpG island region previously studied [27], [39], [40].

Although there are some differences between this study and previously published studies, we believe this work is more robust and comprehensive, as ours employs the largest patient cohort, is population-based, and includes a control group of non-treated CRC patients for comparison. The biological role and timing of *IGFBP3* methylation in CRC is poorly understood; however, recent *in vitro* studies have reported that methylation-induced silencing of *IGFBP3* may lead to significant resistance to cisplatin treatment in lung cancer [42], [43]. Conversely, it is also possible that this event is merely a consequence of CIMP, and *IGFBP3* methylation consequently serves as a surrogate marker of this phenotype and may be an important determinant of poor responses to 5-FU-based adjuvant chemotherapy in CRC, as previously shown by our laboratory [7]. It is very likely that *IGFBP3* methylation alone may not provide requisite sensitivity and specificity for a clinically viable test, and inclusion of additional biomarkers may further improve the clinical significance of this biomarker. Although work from our laboratory and other laboratories have previously provided data for the functional role of *miR-137* and *IGFBP3* methylation in colorectal cancer [21], [44], additional *in-vitro* and *in-vivo* studies in this regard may further facilitate in gathering a better understanding of the biological roles of these biomarkers in colorectal carcinogenesis.

In summary, this study performed a comprehensive analysis of a large, population-based CRC cohort and clearly highlighted the potential roles of the methylation status of *miR137* and *IGFBP3* as diagnostic biomarkers in CRC patients. In addition, our data revealed that *IGFBP3* promoter methylation serves as an independent prognostic biomarker in stage II and III colorectal cancer patients. Our results also suggest that stage II&III CRC patients with high levels of *IGFBP3* methylation do not benefit from adjuvant 5FU-based chemotherapy.

## Supporting Information

Figure S1 **Representative pyrograms of methylation markers in the sept9, twist1, alx4, igfbp3, gas7, and mir137.**
(DOCX)

Information S1 **Supplementary patients and methods.**
(DOCX)

Table S1 **Clinicopathological and molecular features of epicolon-i patients.**
(DOCX)

Table S2 **Primer sequences for pyrosequencing.**
(DOCX)

Table S3 **Clinicopathological features associated with gene methylation.**
(DOCX)
