# Supplementary material for: IGFBP3 Methylation Is a Novel Diagnostic and Predictive Biomarker in Colorectal Cancer
Source: PLoS One. 2014 Aug 15;9(8):e104285. doi: 10.1371/journal.pone.0104285 (PMC4134211; doi:10.1371/journal.pone.0104285)

**Figure S1.** Representative pyrograms of methylation markers in the SEPT9, TWIST1, ALX4, IGFBP3, GAS7, and miR137.


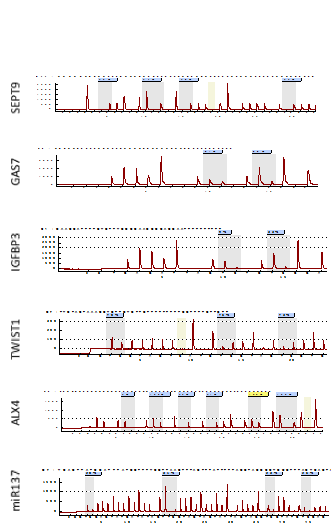

Supplement: Figure S1 — Representative pyrograms of methylation markers in the sept9, twist1, alx4, igfbp3, gas7, and mir137. (DOCX) [file pone.0104285.s001.docx]
