# Supplementary material for: IGFBP3 Methylation Is a Novel Diagnostic and Predictive Biomarker in Colorectal Cancer
Source: PLoS One. 2014 Aug 15;9(8):e104285. doi: 10.1371/journal.pone.0104285 (PMC4134211; doi:10.1371/journal.pone.0104285)
Supplement: Information S1 — Supplementary patients and methods. (DOCX) [file pone.0104285.s002.docx]

**Supporting information: Supplementary Patients and Methods**

**Patients**

This study included 425 non-selected CRC patients that were enrolled as part of the Epicolon project. Epicolon-I is a retrospective observational study of a total of 1222 CRC patients diagnosed in 20 Spanish hospitals between November 2000 and October 2001. 750 out of 1222 had 5 year follow-up data available. The study was approved by the institutional ethics committee of each participant hospital and written informed consent was obtained from all patients. Adjuvant chemotherapy was administered according to standard clinical criteria following standard schedules and doses. Oncologists who decided to administer adjuvant treatment were blinded to the molecular phenotype status (MMR or CIMP tumor status). Mean follow-up (±SD) was 1212 ±717 days (3.4 ±1.9 years).The clinicopathological and molecular features of patients included in those studies are described in Supplementary Table 1 (**Table S1**). Characteristics of Epicolon-I Clinicopathologic features.

**Methods**

**Pyrosequencing (SEPT9, TWIST1, ALX4, IGFBP3 GAS7 and miR137)**

We used bisufite pyrosequencing for quantitative methylation analysis (PSQ HS 96A pyrosequencing system, QIAGEN) in bisulfite modified genomic DNA (EZ DNA methylation Gold Kit, Zymo Research). (Supplementary Figure 1). Primers used were designed using PyroMark 1.0 design software, and short assays to assess methylation in DNA from FFPE were performed within specific CpG island regions in the promoter regions of genes (Supplementary Table 3). PCR conditions were as follows: 15 min 94° C for activation and then 48 cycles consisting of 30 sec 94°C, 30 sec 54 °C and 30 sec 72 °C for SEPT9 promoter assay, TWIST1 assay conditions were the same except for the annealing temperature at 54 °C. 54 °C was also the annealing temperature for ALX4, 56 °C for IGFBP3 and GAS7, and 53 °C for the miR137 promoter assay. Mean percentage of methylation for all the CpG sites included in each assay was calculated for each marker.

**IGFBP3 qMSP**

We performed IGFBP3 quantitative MSP (qMSP) assays (Figure 6) located at the promoter/exon1 CpG island in order to compare methylation values with bisulfite pyrosequencing. The primers and PCR conditions were previously described (1). The COL2A gene was used as a housekeeping reference, and SYBR green analysis was performed on an ABI Prism 7000 instrument using the standard protocol in the manufacturer’s instructions. The percentage of methylated reference (PMR) or degree of methylation at a specific locus was calculated with the IGFBP3:COL2A1 ratio of a sample divided by the IGFBP3:COL2A1 ratio of the completely methylated DNA control.
