# Supplementary material for: IGFBP3 Methylation Is a Novel Diagnostic and Predictive Biomarker in Colorectal Cancer
Source: PLoS One. 2014 Aug 15;9(8):e104285. doi: 10.1371/journal.pone.0104285 (PMC4134211; doi:10.1371/journal.pone.0104285)
Supplement: Table S1 — Clinicopathological and molecular features of epicolon-i patients. (DOCX) [file pone.0104285.s003.docx]

**Table S1.** Clinicopathological and molecular features of Epicolon-I patients.

|  |  | **Epicolon-I patients (425)** |
| --- | --- | --- |
| **Age** | Mean (SD) | 70.4 (11.65) |
| **Age**  **<50**  **>50** | N (%) | 28 (6.7)  390 (93.3) |
| **Gender**  **Male**  **Female** | N (%) | 252 (60.3)  166 (39.7) |
| **Location**  **Proximal**  **Distal**  **Rectum** | N (%) | 117 (27.9)  151 (36.2)  150 (35.9) |
| **MSI**  **MSI-high**  **MSS** | N (%) | 35 (8.2)  425 (91.8) |
| **CIMP**  **Yes**  **No** | N (%) | 90 (29.7)  213 (70.3) |
| **BRAF**  **Mutated**  **No mutation** | N (%) | 37 (11.7)  280 (88.3) |
| **Grade Differentiation Well**  **Poor** | N (%) | 353 (91.7)  22 (8.3) |
| **Mucinous histology**  **Yes**  **No** | N (%) | 52 (12.5)  364 (87.5) |
| **TNM (Stage)**  **I**  **II**  **III**  **IV** | N (%) | 53 (12.8)  140 (33.7)  165 (39.7)  57 (13.7) |
| **Follow-up** Mean | (SD) | 1156 (741) |
