# Supplementary material for: IGFBP3 Methylation Is a Novel Diagnostic and Predictive Biomarker in Colorectal Cancer
Source: PLoS One. 2014 Aug 15;9(8):e104285. doi: 10.1371/journal.pone.0104285 (PMC4134211; doi:10.1371/journal.pone.0104285)
Supplement: Table S2 — Primer sequences for pyrosequencing. (DOCX) [file pone.0104285.s004.docx]

**Table S2**. Primer sequences for pyrosequencing

| **Genes** | **Forward** | **Reverse** | **Pyro-primer** |
| --- | --- | --- | --- |
| ***SEPT9*** | AGAGAATTTTGTTTGGTTGTTTAAATATAG | AAAAAAAATTCCTCCCCTTCC-B | TGTTTAAATATAGTTTTTTGTA |
| ***TWIST1*** | TTTTGGGTTGGTATTGTTAGTTGT | ATAAAAAAAAACCCAATCCATAAAA-B | GGTATTGTTAGTTGTTAGGG |
| ***IGFBP3*** | GTTGAGAAGTAAGTTTGGAAAGGG | ATCCCCAAAAACCCACAAATA-B | TGAGAAGTAAGATTGGAAA |
| ***GAS7*** | TTGGTAATGTTTGTTGATTGATTATATGAG-B | ACCCAAAATACCAACCCTCC | ACCCAAAATACCAACC |
| ***ALX4*** | TGTTGTTGTTAGGGATGAGAG | ACACCCCAATCCAAAAATCTAC | AGGGATGAGAGGTTG |
| ***miR137*** | TGGATTTTTTTTTAGGGAAAT | CCACCAAAACTCTTACTACTC | ATTTTTTTTTAGGGAAAT |
