# Supplementary material for: IGFBP3 Methylation Is a Novel Diagnostic and Predictive Biomarker in Colorectal Cancer
Source: PLoS One. 2014 Aug 15;9(8):e104285. doi: 10.1371/journal.pone.0104285 (PMC4134211; doi:10.1371/journal.pone.0104285)
Supplement: Table S3 — Clinicopathological features associated with gene methylation. (DOCX) [file pone.0104285.s005.docx]

**Table S3.** Clinicopathological features associated with gene methylation

| **Genes** | **Age**  **Mean Methylation (%)**  **(±SD)** | | | **Gender**  **Mean Methylation (%)**  **(±SD)** | | | **TNM Stage**  **Mean Methylation (%)**  **(±SD)** | | | **Location**  **Mean Methylation (%)**  **(±SD)** | | |
| --- | --- | --- | --- | --- | --- | --- | --- | --- | --- | --- | --- | --- |
|  | ≤ 65 | > 65 | P-value | Male | Female | P-value | I +II | III+IV | P-value | Left  colon | Right colon | P-value |
| ***SEPT9*** | 23.6  (±21.8) | 23.8  (±19.8) | NS | 25.6  (±24.39) | 20.9  (±18.6) | **< 0.05** | 24.5  (±20.9) | 22.8  (±19.6) | NS | 24.9  (±20.8) | 20.9  (±18.5) | NS |
| ***ALX4*** | 34.4  (±19.7) | 37.41  (±19.4) | NS | 37.8  (±19.1) | 34.5  (±20.1) | NS | 35.8  (±20.1) | 37.4  (±18.9) | NS | 36.6  (±19.3) | 36.4  (±20.2) | NS |
| ***TWIST1*** | 48.1  (±17.7) | 51.6  (±18.7) | NS | 51.6  (±18.7) | 49.2  (±18.2) | NS | 51.96  (±18.5) | 49.2  (±18.5) | NS | 51.53  (±18.7) | 48.2  (±17.7) | NS |
| ***IGFBP3*** | 42.6  (±16.8) | 45.9  (±17.4) | NS | 45.5(±  17.3) | 44.5  (±17.5) | NS | 45.9  (±17.6) | 43.8  (±17.2) | NS | 44.1  (±17.3) | 47.9  (±17.4) | 0.06 |
| ***GAS7*** | 49.9  (±15.9) | 50.6(±  16.2) | NS | 49.9  (±15.9) | 50.5  (±16.1) | NS | 51.4  (±16.4) | 48.5  (±15.4) | NS | 49.7  (±16) | 51.3  (±16.1) | NS |
| ***miR137*** | 32.24  (±17.8) | 36.5  (±17.3) | **0.05** | 36.4  (±17.9) | 33.6  (±18.5) | NS | 35.8  (±19.1) | 34.6  (±17.1) | NS | 33.4  (±18.4) | 35.1  (±17.9) | NS |
